# Acceptability and understanding of front-of-pack nutritional labels: an experimental study in Mexican consumers

**DOI:** 10.1186/s12889-019-8108-z

**Published:** 2019-12-30

**Authors:** Jorge Vargas-Meza, Alejandra Jáuregui, Alejandra Contreras-Manzano, Claudia Nieto, Simón Barquera

**Affiliations:** Center for Nutrition and Health Research, National Institute of Public Health, 62100 Cuernavaca, Mexico

**Keywords:** Front-of-pack label, Guideline daily allowance, Multiple traffic light, Chilean nutrition policy

## Abstract

**Background:**

Front-of-package labelling is a cost-effective strategy to help consumers make healthier choices and informed food purchases. The effect of labels is mediated by consumer understanding and acceptability of the label. We compared the acceptability and understanding of labels used in Latin-America among low- and middle-income Mexican adults.

**Methods:**

Participants (*n* = 2105) were randomly assigned to one of three labels: Mexican Guideline Daily Allowances (GDA), Ecuador’s Multiple Traffic Lights (MTL), or Chile’s Warning Labels (WL) in red. Label acceptability was evaluated through items regarding likeability, attractiveness and perceived cognitive workload. Objective understanding was evaluated by asking participants to select the product with the lowest nutritional quality among three products. We measured the time participants took to choose the product. Differences in label acceptability, understanding and time required to choose a product across labels were tested.

**Results:**

Compared to the GDA, a higher proportion of participants liked the MTL and WL, considered them attractive, and with a lower perceived cognitive workload (*p* < 0.05). Participants had 4.00 (2.86–5.59) times the odds of correctly identifying the product with the lowest nutritional quality when using the MTL label and 4.52 (3.24–6.29) times the odds when using the WL, in comparison to the GDA. Time required to choose the product was lower for the MTL (Median: 11.25 s; IQR = 8.00–16.09) and the WL (Median = 11.94 s, IQR = 8.56–16.52) compared to the GDA (Median: 15.31 s; IQR = 10.81–20.21; *p* < 0.05). No differences were observed between the MTL and the WL.

**Conclusions:**

GDA had the lowest acceptability and understanding among the labels tested. The MTL and the WL were more accepted and understood, and allowed low- and middle-income consumers to make nutrition-quality related decisions more quickly. WL or MTL may foster healthier food choices in the most vulnerable groups in Mexico compared to the current labelling format.

## Background

Chronic noncommunicable diseases related to diet, such as obesity, type 2 diabetes and cardiovascular diseases, are responsible for 60% of the deaths in the world [1]. In particular, the Latin-American region has experienced a rise in non-communicable diseases posing new challenges to health systems [2]. As a response, several international organizations recommend improving dietary intake at the population level, by limiting the consumption of fats, sugars and salt [3–7]. Front-of-package (FOP) labelling, a system that provides simplified nutrition information on the FOP’s, is a cost-effective strategy to help consumers make healthier choices and informed food purchases and reduce the risk for chronic diseases at the population level [8]. The World Health Organization, as well as other international agencies, have sought to include FOP labelling strategies as part of a comprehensive policy response to the increasing prevalence of obesity and other non-communicable diseases [9].

To date, a growing number of countries in Latin-America have implemented FOP labelling strategies, generally based on local scientific evidence. For example, due to the increase of excess weight in Chilean children, in 2016 the Ministry of Health implemented a new labelling policy in which packaged foods and beverages exceeding specified limits of sodium, sugar, energy and saturated fats had warning label [10]. Studies among mothers and minors have shown that WL are understood and considered to a large extent during the selection of new products [11, 12]. During 2017, various organizations and institutions in Brazil lobbied for a warning system proposal, which also considered warnings for artificial sweeteners and trans fats, in alignment with the nutrient profile model of the Pan American Health Organization (PAHO) [13, 14]. In 2014, Ecuador introduced a MTL labelling [15, 16], indicating in colored bars and letters the amounts of sugar, fat and salt in processed foods. In Mexico the GDA were implemented as a voluntary label in 2011, and in early 2016 they became the mandatory FOP label, along with more than 5 million USD invested in national communication and educational campaigns [17]. However, the implementation of this labelling was not based on the best available evidence [16, 18]. Studies have shown that the Mexican population does not understand this labelling because of the complex quantitative format [19–21]. Thus, the regulation of FOP labels in the country is currently being revised, to replace this label with the most effective format for the Mexican population.

According to Grunert and Wills, for nutrition labels to have any effect, consumers must be exposed to them and must perceive them; then, the effect will be mediated by consumer understanding, as well as by the acceptability of the label [22]. Consistent evidence indicates that label understanding is lower among low-income and low-education populations [23], who, at the same time, are the most nutritionally at risk [24], and the most representative in the Latin-American region. Literature indicates that interpretive formats are the most effective in helping consumers make healthier food choices [22]. Most studies in Latin-American consumers recommend the implementation of WL, based on studies testing the objective understanding of the labels among children and adults [20, 25]. Similarly, studies in Ecuador suggest that MTL are identified by most Ecuadorians and evaluations during the year of their implementation (end of 2015) showed that the label was effective in decreasing the consumption of products labeled in red [26].

Additionally, consumers generally have limited time to process nutritional information provided in nutritional labels. Evidence has shown that time required to make decisions can be as short as 0.04 s in simple tasks (e.g. choosing one’s favorite food), and up to 18 s for more complex tasks [27, 28]. Therefore, effective FOPL should allow consumers make informed purchases in less than 30 s [19, 21]. However, to date few studies have focused on testing the acceptability and time required to make an informed decision using these labels among Latin-American consumers [20, 29].

In order to provide evidence to support policies aiming to implement effective and equitable FOP labelling systems at the national and regional level and generate academic and political discourse globally, we aimed to compare the acceptability and understanding of the three FOP labels used in Latin-America, among low- and middle-income Mexican adults using an experimental study.

## Methods

### Study design

From May to June 2018, we developed an online three-arm unblinded randomized experiment. We used an adaptation of the model proposed by Grunert and Wills to study the effects of nutrition labels on consumers [22]. This model states that for nutrition labels to have any effect, consumers must be exposed to them and must perceive them. The effect will then be mediated by consumer understanding, as well as by the acceptability of the label.

This study is part of an online shopping simulation trial testing the effect of FOP labels on shopping intentions. After the shopping simulation, we employed a web-based tool to evaluate the acceptability and objective understanding of the assigned label. This study reports the results of this latter part of the trial. The ethics and research committees of the Mexican National Institute of Public Health approved the study protocol and instruments.

### Recruitment and procedures

A convenience sample of adults (> 18 years old) was used for this study. Trained undergraduate student research assistants from eight universities across the country recruited the study participants. Two members of our research team (JVM and ACM) trained research assistants on how to approach and recruit participants and obtain informed consent. Research assistants were instructed to recruit at least 20 participants each, in any of the predefined public places. Places were selected by convenience by our research team, based on their use by low- and middle-income groups in Mexico. These places included public schools, public squares, public health centers, as well as supermarket chains and shopping centers located in low-income neighborhoods. At each place, research assistants approached all potential participants and, after explaining study objectives, invited them to be part of the study.

Then, research assistants accessed a unique web address where our web-based tool was hosted using a tablet or laptop with internet-access, and individuals were screened for eligibility using a 3-item screener. Only adults (> 18 years old) consuming at least one of the five food groups included in the shopping site (salty snacks, beverages, dairy products, breakfast cereals and ready-made foods) and who shopped for groceries at least twice per week were eligible. If the potential participant or any of their direct family members worked in the food and beverage industry, they were excluded from the study. The web-based tool automatically informed research assistants if the participant was eligible. In such case, informed written consent was obtained. Then, research assistants handed participants the tablet or laptop, where participants completed a demographic and health survey, and accessed an online shopping site to simulate a shopping situation. After the shopping simulation, participants answered a questionnaire related to the acceptability of the assigned label and then they were required to do some exercises designed to test the objective understanding of the label.

### Sample size determination and sampling procedure

Based on previous studies [30], and considering a significance level of 0.05 and a power of 80%, we estimated that a total of 832 participants were needed in each label group to detect a difference of 6.2 percentage points (the smallest difference reported) in the proportion of participants reporting to like the GDA versus the MTL. Considering three label groups, the ideal total sample size was determined to be of 2496 participants.

**Fig. 1:**
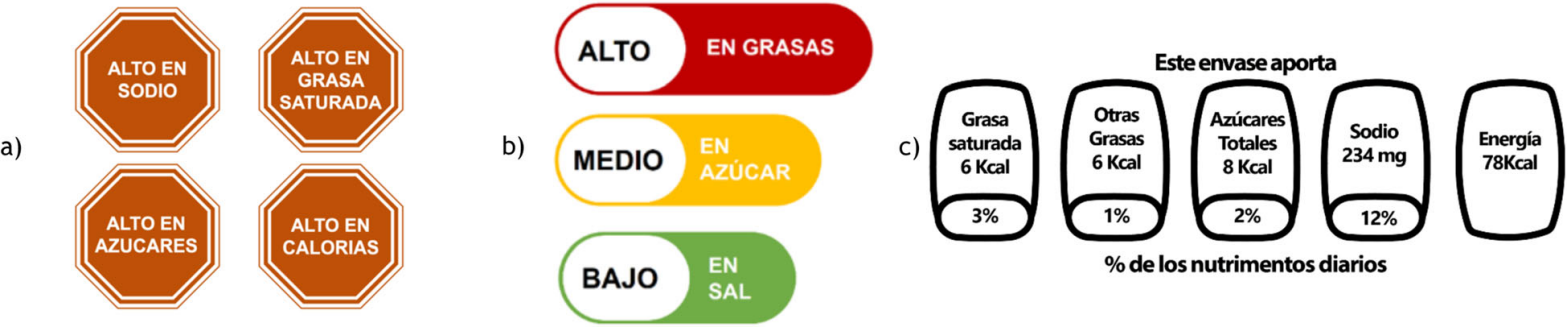
Front-of-pack label assigned to participants. **a** Warning Label in Red, **b** Multiple Traffic Light and **c** Guideline Daily Allowance

Before entering the online shopping site, our web-based tool automatically assigned participants to one of three FOP labels (Fig. 1) using a simple randomization algorithm, blinding the research assistants to the assigned condition:

1. Mexican GDA, indicating the grams and percentages (according to the guideline-based daily intakes) per portion of kilocalories, saturated fats, other fats, sugars, and sodium. The GDA label was the control group because they are required to appear on the FOP of food products in Mexico.
2. Ecuadorian Multiple Traffic Lights (MTL), color coded (red, yellow, and green, respectively), including text descriptors to indicate high, medium, or low content of total fat, sugar and salt.
3. Chilean Warning Labels (WL) in red, indicating when a product exceeds the content of energy, sodium, total sugar and saturated fat. We decided to put the WL in red because previous work by our research group demonstrated this color increased label acceptability [31].

Blinding of participants was not possible given the nature of the intervention. After completing the demographic and health survey and being assigned to a group, participants were required to view a video explaining how to correctly interpret the assigned label. These videos corresponded to official videos used in each country (i.e. Mexico, Ecuador and Chile) to promote the correct use of the current labelling.

### Acceptability of FOPL

Label acceptability was evaluated using three indicators: likability, attractiveness and perceived cognitive workload. These dimensions were based on the framework of system acceptability developed by Nielsen [32], which have been previously used to explore acceptability of FOP labels in French consumers [30]. Participants were asked to rate the assigned label using 10 statements, adapted from recent FOPL studies in French consumers [30]. To evaluate likability of the label, consumers were asked to rate their agreement with the following statements: 1) “I like this label”, 2) “I want to see this label on the FOP’s”, and 3) This label will help me choose a healthier product”. To evaluate the attractiveness of the label the following statements were used: 1) “This label does not catch my attention”, 2)“This label provides me with the information I need”, 3) “This label is easy to identify”, 4)” This label provides reliable information”. Perceived cognitive workload was evaluated with the following statements: 1) “This label is too complex to understand”, 2) “This label takes too long to understand”, and 3) “This FOPL makes me uncomfortable” [30]. Five response options were included: 1) Strongly agree, 2) Agree, 3) Neither agree nor disagree, 4) Disagree, and 5) Strongly disagree. For each statement, participants choosing options 1) and 2) were classified as agreeing, whereas those choosing options 3), 4) or 5) were classified as disagreeing.

### Objective understanding

We tested the objective understanding of FOPL with a series of five exercises per participant, corresponding to the five food categories included in the online supermarket (sugary drinks, salted snacks, cereals, dairy products and ready-to-eat foods). Participants were asked to choose the product with the lowest nutritional quality among three products of the same food category.

For this purpose, we used the same set of 60 products used in the online shopping site (12 products for each food category), all of which are normally bought on a weekly basis. These products were selected from a database collected by the Mexican National Institute of Public Health between 2015 and 2016 [33]. We used the Nutrient Profiling Scoring Criterion (NPSC) model to assign all foods a healthfulness score and select a variety of products ranging in nutritional quality [34]. The nutritional quality and nutritional content of the foods included in the virtual supermarket is shown in Additional file 1. Underlying nutrition criteria for each label were used to assign the corresponding label to food products [10, 26, 35]. However, the criteria for classifying nutrient content as high in the MTL and the WL were based on 2016 Chilean regulations [10].

**Fig. 2:**
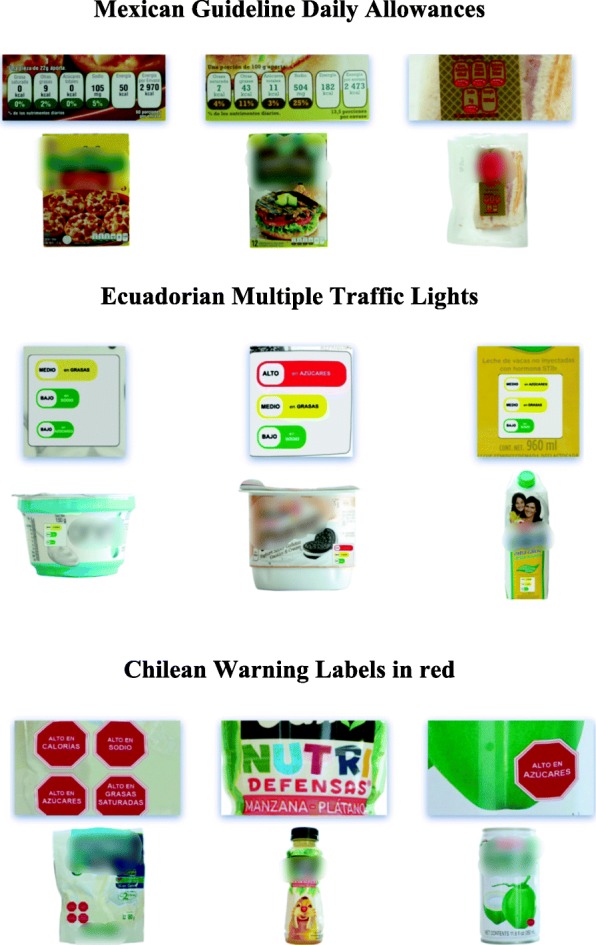
Example of how the three products were displayed, by labelling condition

All exercises followed the same dynamic. Participants were shown 3 products ranging in nutritional quality, randomly selected from the list of 12 products of the same food category. The image of each product displayed the label on the FOP. Labels were positioned in the lower right corner of the FOP, covering roughly the same surface area. Additionally, the amplified image of the label was shown on the top of each product. Fig. 2 shows how products were displayed for each of the labelling conditions. Participants were instructed to drag and drop the product with the lowest nutritional quality in a box area in less than 30 s. This procedure was repeated for each one of the five food categories. In order to prevent any ordering effects, we also randomized the order in which the food categories were presented.

### Time required to identify the product with the lowest nutritional quality

The time participants required to identify the product with the lowest nutritional quality was automatically registered by the system.

### Covariates

Information on gender, age, monthly income per household, education level, occupation, body mass index (BMI), presence of chronic conditions, and nutrition knowledge was collected. Only included close-ended questions were used, taken from previously validated surveys with pre-established responses [36].

### Data analysis

We tested randomization of observable demographic and health characteristics of participants by comparing variables between experimental groups using Chi-squared tests (for categorical variables) and linear regression models (for continuous variables). Randomization successfully created equivalent groups on all demographic characteristics, so subsequent analyses did not control for these variables. Since time variables were skewed, medians and interquartile ranges were estimated.

To evaluate the acceptability of the labels, we estimated the proportion of participants agreeing to each of the 10 acceptability statements. Differences between proportions were estimated using Chi-squared tests.

To compare the understanding of the assigned labels, we estimated the proportion of participants and odds of correctly identifying the least healthy option in all food categories and across food categories. Differences in objective understanding across demographic characteristics (i.e. gender, age category, household income, education level and nutrition knowledge) were also estimated by introducing an interaction term between the label group and the characteristic of interest (e.g., Label group X age category).

To test differences in the median time required to choose the product with the lowest nutritional quality, we used median regression models introducing the assigned label as the independent variable. Models were run for all food categories and across food categories.

GDA was considered the reference category in all models. Comparisons between the MTL and the WL were made using post-estimation tests. Statistical assumptions were verified before running all tests and models, accordingly. All tests of significance were two-sided, and a *p*-value < 0.05 was considered significant. Stata version 14 was used for the analysis.

## Results

Of 2946 potential participants who accepted to take part in the study, 841 were not eligible, leaving a total of 2105 (GDA = 697, WL = 708, MTL = 700) participants who started and completed the study. No differences were observed in demographic and health characteristics between label groups (*p* > 0.05) (Table 1). Participants had a mean age of 37.6 (±16.6) years, were mostly females (56.7%), were married (40.6%), and more than half were diagnosed with a chronic disease (8.5% Diabetes, 12.6% Hypertension, 22.2% Dyslipidemia). Almost 78% reported to be very interested in their health, and more than 50% reported having little or no knowledge of nutrition. Almost half of the participants (48.9%) had a monthly income of less than $ 6800 Mexican pesos (≈$357 USD), which is similar to the average income of the fifth decile of socioeconomic status in Mexico [37], and most reported having an educational level at or below high school (51.5%).

**Table 1:** Participants’ demographic characteristics, health interest, and nutrition knowledge by assigned label (*n* = 2105)

	GDA (*n* = 697)	MTL (*n* = 708)	WL (*n* = 700)	*p*-value
	*n* (%)	*n* (%)	*n* (%)	
Age				
18- 29y	322(46.2)	324 (45.8)	329 (47.0)	0.904
30-49y	192 (27.6)	193 (27.3)	178 (25.4)	
> 50y	183 (28.3)	191 (26.7)	193 (27.6)	
Gender				
Female	400 (57.4)	416 (58.8)	381 (54.4)	0.246
Marital status				
Single	251 (36)	261 (36.9)	254 (36.3)	0.333
Married/ living with a partner	352 (52.3)	370 (52.3)	351 (50.1)	
Divorced	67 (9.6)	44 (6.2)	59 (8.4)	
Widower	27 (3.9)	33 (4.7)	36 (5.1)	
Education				
Elementary school or less	50 (7.2)	45 (6.4)	52 (7.4)	0.901
Secondary School	89 (12.8)	90 (12.7)	74 (10.6)	
High school	217 (31.1)	230 (32.5)	236 (33.7)	
Graduate/ Postgraduate	341 (48.9)	343 (48.6)	338 (48.3)	
Household income				
< $2699	123 (17.7)	122 (17.2)	121 (17.3)	0.989
$2700-6799	218 (31.3)	222 (31.4)	224 (32.0)	
$6800-11,599	187 (26.8)	190 (26.8)	183 (26.1)	
$11,600-34,999	120 (17.2)	124 (17.5)	131 (18.7)	
> $35,000	49 (7.0)	50 (7.1)	41 (5.9)	
Occupation				
Unemployed/ Other	46 (6.7)	54 (7.6)	70 (10.0)	0.510
Student	164 (23.5)	168 (23.7)	150 (21.4)	
Housemaid/ Home maker	146 (21.0)	134 (18.9)	136 (19.3)	
Employee	280 (40.2)	293 (41.4)	284 (40.7)	
Salesman/woman	61 (8.8)	59 (8.3)	50 (8.6)	
Previous diagnosis of chronic disease				
Diabetes	57 (8.2)	61 (8.6)	59 (8.4)	0.957
Hypertension	90 (12.9)	86 (12.2)	89 (12.7)	0.904
Overweight	171 (24.5)	161 (22.7)	177 (25.3)	0.518
Obesity	66 (9.5)	66 (9.3)	67 (9.6)	0.987
Hypercholesterolemia	79 (11.3)	80 (11.3)	80 (11.4)	0.997
Hypertriglyceridemia	68 (9.8)	75 (10.6)	84 (12.0)	0.393
Health interest				
Not interested/ A Little interested	143 (20.5)	145 (20.5)	169 (24.1)	0.161
Very interested	554 (79.5)	563 (79.5)	531 (75.9)	
Self-reported nutrition knowledge				
Not knowledgeable	148 (21.2)	149 (21.1)	151 (21.6)	0.998
A little knowledgeable	292 (41.9)	295 (41.7)	294 (42.0)	
Somewhat knowledgeable/ Very knowledgeable	257 (36.9)	264 (37.3)	255 (36.4)	

*GDA* Guideline Daily Allowance, *MTL* Multiple Traffic Light, *WL* Warning Labels. Chi2 was used to test for significant differences between labelling conditions

### Label acceptability

The results of the acceptability of the labels is presented in Table 2.

**Table 2:** Proportion of participants who strongly agreed or agreed to the statements evaluating label acceptability (*n* = 2105)

	GDA (*n* = 697)	MTL (*n* = 708)	WL (*n* = 700)
	*n* (%)	*n* (%)	*n* (%)
Liking			
I like this label	296 (42.5)	**655 (92.5)**	**557 (79.6)**^**a**^
I want to see this label on the front of packages	380 (54.5)	**644 (91.0)**	**582 (83.1)**^**a**^
This label will help me choose a healthier product	328 (47.1)	**636 (89.8)**	**585 (83.6)**^**a**^
Attractiveness			
This label does not catch my attention	446 (64.0)	**145 (20.5)**	**228 (32.6)**^**a**^
This label provides me with the information I need	360 (51.7)	**588 (83.1)**	**540 (77.1)**
This label is easy to identify	379 (54.4)	**667 (94.2)**	**623 (89.0)**^**a**^
This label provides reliable information	380 (54.5)	**564 (79.7)**	**531 (75.9)**
Perceived cognitive work-load			
This label is too complex to understand	438 (62.8)	**90 (12.7)**	**116 (16.6)**^**a**^
This label takes too long to understand	431 (61.8)	**77 (10.9)**	**112 (16.0)**^**a**^
This label makes me uncomfortable	351 (50.4)	**61 (8.62)**	**107 (15.3)**^**a**^

*GDA* Guideline Daily Allowance, *MTL* Multiple Traffic Light, *WL* Warning Labels

**Bold** numbers indicate significant difference (*p* < 0.05) with GDA. a: Significant difference (*p* < 0.05) between MTL and WL

Chi^2^ was used to test for significant differences between labelling conditions

#### Likability

Around 90% of participants assigned to the MTL strongly agreed or agreed they liked the label, wanted to see the label on the front of food packages, and considered that the label would help them choose a healthier product. These numbers were slightly lower (≈80%) for participants assigned to the WL (*p* < 0.05), and considerably lower (≈50%) for those assigned to the GDA (p < 0.05).

#### Attractiveness

Compared to the GDA, the MTL and the WL led to a higher proportion of participants considering the label provided them with the information they needed, was easy to identify, and provided reliable information (*p* < 0.05). Conversely, the GDA led to the highest proportion of participants reporting that the label did not catch their attention, compared to the MTL and the WL (p < 0.05).

#### Perceived cognitive workload

Compared to other labels, the GDA led to a higher proportion of participants considering the label was too complex, took too long to understand, or it made them uncomfortable (p < 0.05). In contrast, compared to other labels, the MTL was the label with the least perceived cognitive workload, with less than 15% of participants of this group agreeing or strongly agreeing with the statements. (*p* < 0.05).

### Objective understanding

**Fig. 3:**
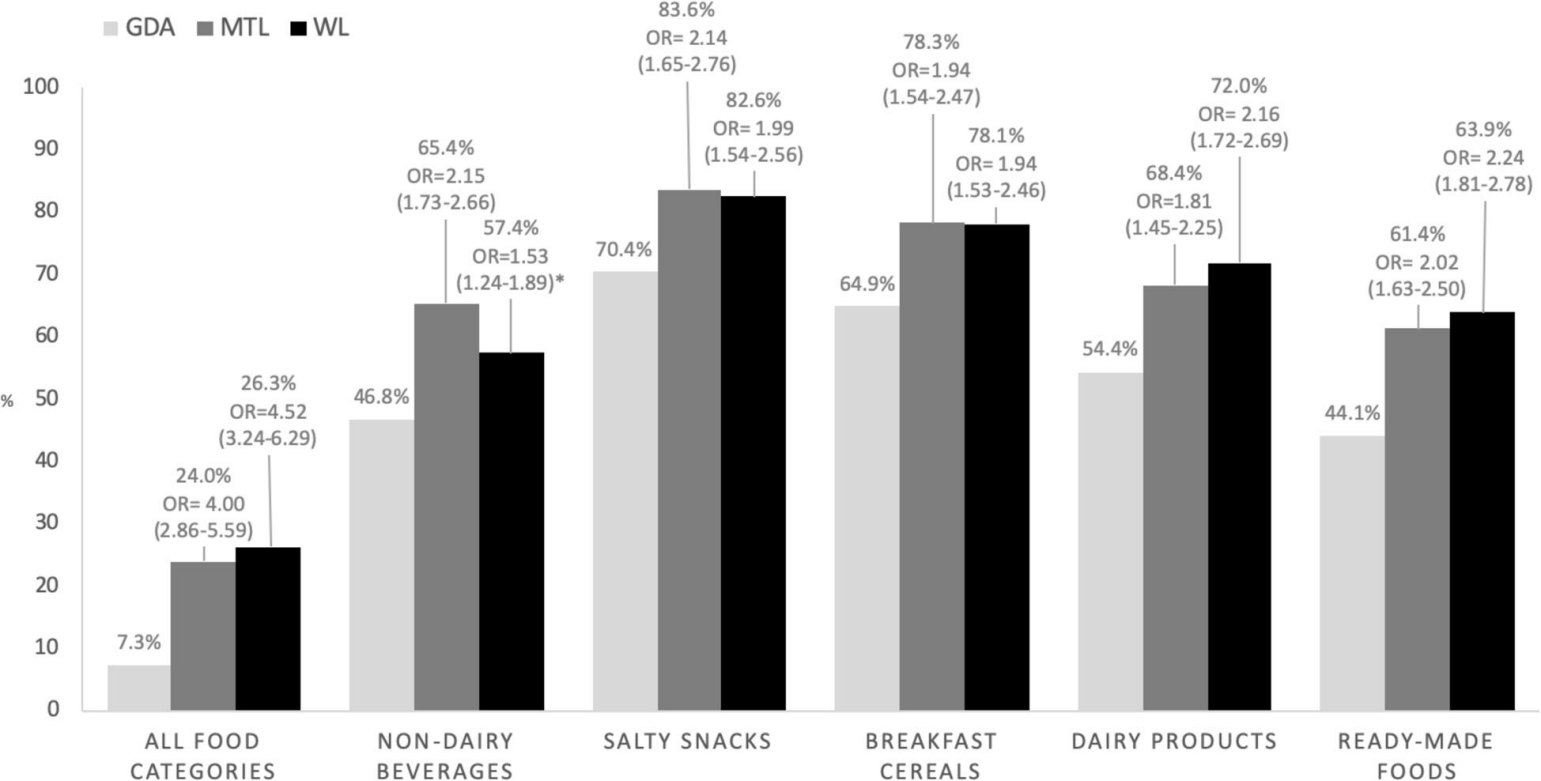
Proportion and odds for correctly identifying the product with the lowest nutritional quality. GDA: Guideline Daily Allowance; MTL: Multiple Traffic Light, WL: Warning Labels. *Statistically significant differences (*p* < 0.05) between MTL and WL

The proportion of participants correctly identifying the product with the lowest nutritional quality was highest for the MTL and the WL in all food categories and across them (Fig. 3). When considering all food categories, participants had 4.00 (2.86–5.59) times the odds of correctly identifying the product with the lowest nutritional quality when using the MTL label and 4.52 (3.24–6.29) times the odds when using the WL, in comparison to the GDA (*p* < 0.05). When exploring the objective understanding of the label across food categories, participants in the MTL and the WL were about twice as likely to correctly identify the product with the lowest nutritional quality, compared to the GDA (p < 0.05). No differences were observed between the WL and the MTL, except for non-dairy beverages where the MTL outperformed the WL (p < 0.05).

We did not find differences in label understanding across sociodemographic characteristics (*p* value for all interaction terms > 0.05) (see Additional file 2). Differences in the proportion of participants correctly identifying the least healthy option in all food categories across labels were similar in the full sample and across groups of participants with similar demographic characteristics.

### Time required to identify the product with the lowest nutritional quality

**Fig. 4:**
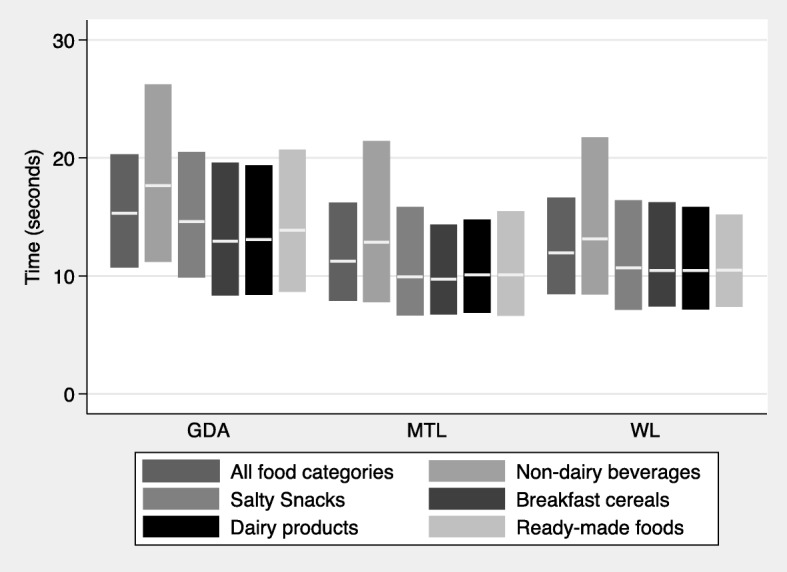
Time required to identify the product with the lowest nutritional quality. GDA: Guideline Daily Allowance; MTL: Multiple Traffic Light, WL: Warning Labels. Box-plots represent medians and interquartile ranges

Fig. 4 presents the median and IQR for the time required to identify the product with the lowest nutritional quality across label groups for all food groups and across food categories. Table 3 presents the regression coefficients for each label group derived from median regression models. Both the MTL and the WL led to shorter median times required to identify the product with the lowest nutritional quality compared to the GDA. Similar results were observed across all food group categories, with the biggest differences for non-dairy beverages.

**Table 3:** Median regression models for the time to identify the product with the lowest nutritional quality

	GDA (n = 697)	MTL (*n* = 708)	WL (*n* = 700)
		β (95% CI)	β (95% CI)
All food categories	Reference	**−4.03 (−4.82, −3.22)**	**−3.36 (− 4.17, −2.56)**
Food categories			
Non-dairy beverages	Reference	**−4.82 (−6.12, − 3.52)**	**−4.52 (− 5.82, − 3.21)**
Salty snacks	Reference	**− 4.64 (− 5.58, − 3.70)**	**− 3.92 (− 4.86, − 2.98)**
Breakfast cereals	Reference	**−3.19 (− 4.10, − 2.27)**	**−2.46 (− 3.37, − 1.54)**
Dairy products	Reference	**−3.00 (− 3.80, − 2.19)**	**−2.61 (− 3.41, − 1.81)**
Ready-made foods	Reference	**−3.78 (− 4.69, − 2.87)**	**−3.39 (− 4.30, − 2.48)**

GDA: Guideline Daily Allowance; MTL: Multiple Traffic Light, WL: Warning Labels

**Bold** numbers indicate significant difference (p < 0.05) with GDA

## Discussion

In this study we aimed to investigate the acceptability and objective understanding of the three FOP labels currently used in Latin-America. Our study showed that among a sample of low- and middle-income Mexican consumers, both the MTL and the WL were more accepted and understood than the GDA, and that these labels required less time to choose the product with the lowest nutritional quality. In general, the MTL had a better acceptability than the WL, however no differences were observed in label understanding and time required to make a nutritional quality decision between these labels.

### Acceptability

In terms of label acceptability, results suggest that the MTL was the most accepted among the three labels [38], followed by the WL. Approximately 90% of participants liked the MTL and considered it had a low perceived cognitive workload. These results are in line with previous work among Mexican consumers showing that the MTL are among the most liked label format in this population [20]. Studies among European consumers have also shown high acceptability of MTL [38]. Warning labels were also highly accepted by participants. To date, few studies have evaluated the acceptability of WL among consumers [39]. The successful implementation of any FOP label partially depends on its acceptability by consumers and policy makers [40]. Nonetheless, evidence from warning labels on tobacco products suggest that the effect of these labels is mediated by elicited negative emotional responses [41, 42]. Therefore, in this study we tested a red version of the WL, since previous qualitative research by our team suggested this change would improve label acceptability [31]. Our results are consistent with studies in Chile and Canada supporting the acceptability of these labels [39, 43]. Importantly, the high acceptability of the MTL and the WL confirms results from other studies suggesting that labels providing positive and negative (i.e. MTL) or only negative (i.e. WL) evaluation of foods do not cause any more discomfort to the consumer compared to those providing a neutral evaluation, such as the GDA [30].

Conversely, our study showed that the acceptability of the Mexican GDA was the lowest, with only approximately half of participants liking the label and considering it attractive, and more than half perceiving it required a high cognitive work load. Since this labelling format was introduced in 2012 as a voluntary label, and in 2016 as the mandatory FOP label in Mexico, the acceptability of the label could be expected to be higher due to the familiarity with the format. In fact, previous studies conducted in Canada and France after implementation of the GDA showed that this label format was the preferred among consumers, possibly due to their familiarity with the label [30, 44]. However, our data does not support this hypothesis. Our results, along with previous studies showing difficulties in the understanding of this labelling format [19–21], confirm that GDA may not be the best labelling format among Mexican consumers.

### Objective understanding

The understanding of a label is a key factor when processing nutrition labels and making purchasing decisions [22]. Our results showed that the MTL and the WL were the most effective in helping consumers identify the product with the lowest nutritional quality, when considering all food categories, as well as across them. These results are in line with a review showing that color-coded labels and the inclusion of text in a labelling format, as in the case of the MTL and the WL, perform better than GDA to compare the nutritional quality of products [45]. Our results are in line with studies among Chilean consumers showing that WL are understood in any age group [11, 25]. Further, studies suggest that WL have also helped consumers understand the underlying driver addressed by the labels. For example, Correa and collaborators showed that one year after implementing this labelling in Chile, mothers with underage children were aware that WL aimed to reduce obesity [46].

In our study we were also able to explore label understanding across different population groups. Results showed that both the MTL and the WL were more effective than GDA across all subgroups studied, including low income and low education participants, who are the most nutritionally at risk [24].

### Time required to identify the product with the lowest nutritional quality

A novel result of our study was the objective measurement of the time required by participants to identify a product of low nutritional quality when using GDA, WL and MTL. Consumers generally have limited time to process nutritional information provided in nutritional labels. Studies have shown that simple labels, as the WL or the MTL, reduce the cognitive effort and time needed to process the information compared to more detailed labels, like the GDA [45, 47, 48]. In line with these results, in this study participants assigned to the MTL and WL required, on average, approximately 11–12 s to choose the product with the lowest nutritional quality, whereas those in the GDA group required more than 15 s. These results are also in line with our results showing a higher perceived cognitive workload for the GDA, compared to the MTL or the WL. Previous scientific evidence has shown that an effective FOPL requires people take between 12 and 30 s to make an informed food selection [19, 21]. However, analysis from the online shopping task executed by these participants before evaluating the acceptability and subjective understanding of the assigned label showed that purchasing decisions were made in no more than 5 s [49]. Studies among consumers have shown that time required to make decisions can be as short as 0.04 s in simple tasks (e.g. choosing one’s favorite food), but when more complex comparisons are required time can increase up to 18 s [27, 28]. There is also evidence suggesting that consumers only glance the nutrition information and do not process the information further at the point of purchase [22], explaining shorter times involved in making purchasing decisions. In this case, participants were required to execute a specific task, which involved inferring the nutritional quality of the product from the information provided on the FOP. By our results it can be inferred that the MTL and the WL helped consumers identify the product with the lowest nutritional quality more quickly. Previous results by our research team have also shown that these labels help consumers make healthier purchasing intentions more quickly [49]. Effective FOP nutritional labels should help summarize all the information in a simple and easy to understand format to influence the customer’s decision-making process. It could be expected that a FOP label that enables making decisions in a shorter time, as the MTL and the WL, will be more effective in helping consumers make healthier purchasing decisions [22].

### Limitations

To our knowledge, this is the first Mexican experimental study that compares the three FOP labels currently used in Latin-America, among low to middle-income participants, who are the most vulnerable for non-chronic diseases and least nutritionally literate. Strengths of this study include the use of an experimental design, ensuring that the influence of confounding from observed and unobserved factors was minimal. We also demonstrated consistent effects across a variety of study outcomes, using objective measures of label understanding and time required to identify the product with the lowest nutritional quality. Despite the former, this study is subject to a variety of limitations. First, the recruitment process was not intended to provide a representative sample of low- to middle-income adults and we were not able to estimate a response rate, which may have biased our results. Therefore, the representativeness of our results is limited to populations with similar characteristics as our sample. Although the sample approximates the demographic profile of the Mexican population in terms of education level and prevalence of diagnosed diabetes and hypertension [50–52], and the distribution of household income is similar to that reported by national estimates [37], the reported prevalence of overweight and obesity as well as dyslipidemias was considerably lower [53, 54]. This may be partially explained by the known under-reporting for weight and over-reporting for height; however, it may also mean that our sample had healthier food patterns and therefore may have been more likely to use labels than the general population. On the other hand, it is also possible that given that our sample was comprised of low- and middle-income participants, their self-perception of overweight or awareness of other chronic conditions was lower compared to the general population [52, 55]. Second, we only tested three types of labels and did not include a no-label condition. This decision was made because GDA is the mandatory label in Mexico. Although including a no-label condition would have provided insightful information for other regions where FOP labels have not been introduced, this study design provides key information for decision-makers in Mexico and contributes to labelling efforts in the region by including some of the most relevant labels in Latin America. Third, although we considered real food products and prices, our study was limited in its ability to replicate the real shopping experience. Therefore, our study provides information on the mediators of the effect of FOP labels (i.e., label acceptability and objective understanding); the effect of the labels on real-life purchasing decisions cannot be inferred from these results. Fourth, images of the products only displayed the FOP, and the potential interaction effects with the nutrition fact panel or the list of ingredients was not captured. However, we used real food products, and no nutritional or health declarations on the FOP were removed, allowing participants to make decisions considering these elements too. Finally, our sample size was smaller than the ideal size (i.e. < 832 participants per label group). This may have limited our ability to identify small differences in the acceptability and understanding of across labels (i.e. differences between the MTL and the WL).

## Conclusions

Results of our study indicate that, despite GDA’s implementation in Mexico since 2011, along with important investments in national communication and educational campaigns, this label format had the lowest acceptability and understanding among the labels tested. Simple labels, such as the MTL and the WL, were more accepted and understood, and allowed low- and middle-income consumers make nutrition-quality related decisions more quickly. Thus, our results confirm the potential of WL or MTL to foster healthier food choices in the most vulnerable groups in Mexico compared to the current labelling format.

## Supplementary information

**Additional file 1 Table S1** Nutritional quality and nutrient content of the food products used. This table provides the mean (min-max) nutritional quality, energy and nutrient (total fat, saturated fat, sugar, fiber and protein) content per 100 g/mL of the products used.

**Additional file 2 Table S2** Proportion of participants correctly identifying the least healthy option in all food categories across demographic characteristics. This table provides descriptive information (proportions and percent’s) of the participants (gender, age, education, household income, nutrition knowledge) according to the assigned frontal labeling.

## Data Availability

The datasets used and/or analyzed during the current study are available from the corresponding author on reasonable request.

## Abbreviations

BMI: Body Mass Index
FOP: Front-of-Package
GDA: Guideline Daily Allowance
MTL: Multiple Traffic Lights
NPSC: Nutrient Profiling Scoring Criterion
PAHO: Pan American Health Organization
WL: Warning Label
