# Supplementary material for: Acceptability and understanding of front-of-pack nutritional labels: an experimental study in Mexican consumers
Source: BMC Public Health. 2019 Dec 30;19:1751. doi: 10.1186/s12889-019-8108-z (PMC6938009; doi:10.1186/s12889-019-8108-z)
Supplement: Supplementary file 2 — Additional file 2 Table S2 Proportion of participants correctly identifying the least healthy option in all food categories across demographic characteristics. This table provides descriptive information (proportions and percent’s) of the participants (gender, age, education, household income, nutrition knowledge) according to the assigned frontal labeling. [file 12889_2019_8108_MOESM2_ESM.docx]

**Additional file 2: Table S2. Proportion of participants correctly identifying the least healthy option in all food categories across demographic characteristics.**

|  | **GDA**  (n= 697) | **MTL**  (n= 708) | | **WL**  (n= 700) | |
| --- | --- | --- | --- | --- | --- |
|  | **%** | **%** | **p-value Interaction term** | **%** | **p-value Interaction term** |
| **Gender** |  |  |  |  |  |
| Males | 6.40 | **26.37** |  | **26.65** |  |
| Females | 8.00 | **22.36** | 0.188 | **25.98** | 0.427 |
| **Age category** |  |  |  |  |  |
| 18- 29y | 8.07 | **23.77** |  | **26.75** |  |
| 30-49y | 7.81 | **24.87** | 0.810 | **25.28** | 0.919 |
| >50y | 5.46 | **23.56** | 0.355 | **26.42** | 0.356 |
| **Education** |  |  |  |  |  |
| Elementary school or less | 4.00 | **20.00** |  | 13.46 |  |
| Secondary School | 4.49 | **18.89** | 0.847 | **31.08** | 0.609 |
| High school | 6.45 | **22.17** | 0.348 | **28.81** | 0.570 |
| Graduate/ Postgraduate | 9.09 | **27.11** | 0.669 | **25.44** | 0.917 |
|  |  |  |  |  |  |
| **Household income** |  |  |  |  |  |
| <$2,699 | 8.13 | **22.13** |  | **28.1** |  |
| $2,700-6,799 | 6.42 | **25.23** | 0.401 | **28.57** | 0.577 |
| $6,800-11,599 | 8.56 | **22.63** | 0.957 | **25.68** | 0.719 |
| $11,600-34,999 | 7.50 | **29.03** | 0.421 | **24.43** | 0.854 |
| >$35,000 | 4.08 | 16.00 | 0.715 | 17.07 | 0.921 |
| **Nutrition Knowledge** |  |  |  |  |  |
| Not knowledgeable | 8.78 | **22.82** |  | **27.15** |  |
| A little knowledgeable | 7.19 | **29.83** | 0.183 | **25.85** | 0.728 |
| Somewhat or very knowledgeable | 6.61 | **18.18** | 0.962 | **26.27** | 0.559 |

GDA: Guideline Daily Allowance; MTL: Multiple Traffic Light, WL: Warning Labels.

**Bold** numbers indicate differences (p<0.05) compared with GDA.
